# Epidemiological, life style, and occupational factors associated with lower limb varicose veins: a case control study

**DOI:** 10.1186/s42506-021-00075-0

**Published:** 2021-07-06

**Authors:** Shahira Elamrawy, Iman Darwish, Sameh Moustafa, Noha Elshaer, Nesma Ahmed

**Affiliations:** 1 Public Health, Preventive and Social Medicine, Community Medicine Department, Faculty of Medicine, Alexandria University, Alexandria, Egypt; 2 Vascular Surgery Department, Faculty of Medicine, Alexandria University, Alexandria, Egypt; 3 Industrial Medicine and Occupational Health, Community Medicine Department, Faculty of Medicine, Alexandria University, Champolion Street, El Azareeta, Alexandria, 21131 Egypt; 4 Ministry of Health, Alexandria, Egypt

**Keywords:** Epidemiological factors, Life style, Occupational factors, Varicose veins

## Abstract

**Background:**

Few data were documented about risk factors for lower limb varicose veins (LLVV) among Egyptian population. Identifying modifiable risk factors is crucial to plan for prevention. The current research aims to study the epidemiological, life style, and occupational factors associated with LLVV in a sample of Egyptian population.

**Methods:**

A case control study was adopted. Cases with LLVV (*n* = 150) were compared with controls (*n* = 150). Data was collected using an interview questionnaire and clinical assessment. Data was analyzed using the univariate and multivariate logistic regression analyses.

**Results:**

According to multivariate analysis among all participants (*n* = 300), the odds of LLVV was 59.8 times greater for those who *frequently lift heavy objects* (95% CI = 6.01, 584.36) and 6.95 times higher for those who *drink < 5 cups of water/day* (95% CI = 2.78, 17.33). Moreover, it was 4.27 times greater for those who *infrequently/never consume fiber-rich foods* (95% CI = 1.95, 9.37) and 3.65 times greater for those who *stand > 4 h/day* (95% CI = 1.63, 8.17). Additionally, odds of LLVV was 3.34 times greater for those who report *irregular defecation habit* (95% CI = 1.68, 6.60), and 2.86 times higher for those who *sleep < 8 h/day* (95% CI = 1.14, 7.16), and 2.53 times higher for *smokers* compared with ex-smokers/non-smokers (95% CI = 1.15, 5.58). In addition, a *standing posture at work* was an independent predictor of LLVV among ever employed participants (*n* = 234) in the current study (OR = 3.10; 95% CI = 1.02, 9.38).

**Conclusions:**

This study highlighted seven *modifiable* independent predictors of LLVV mostly related to the life style, namely, frequent lifting of heavy objects, drinking < 5 cups of water/day, infrequent/no consumption of fiber-rich food, standing more than 4 h/day, irregular defecation habit, sleeping less than 8 h/day, and smoking. These findings provide a basis to design an evidence-based low-cost strategy for prevention of LLVV among Egyptian population.

## Introduction

Chronic venous disease (CVD) is a prevalent condition that tends to worsen with age. Symptoms of CVD include leg pain, discomfort, and heaviness, whereas the clinical signs are varicose veins, edema, skin discoloration, lipodermatosclerosis, and in severe cases, venous ulceration [1]. Lower limb varicose veins (LLVV) may occur as a primary disease as a result of an internal biochemical or morphologic abnormality of the vein wall or as a result of secondary causes such as thrombosis or obstruction of deep veins, superficial thrombophlebitis, arteriovenous fistulas, and pressure on the abdominal veins during pregnancy or from a tumor [2]. Although LLVV seem to be simple cosmetic with benign nature problem, they can be a source of serious complications which can lead to missed work days, lower quality of life, and even loss of a limb or life [3].

Globally, according to Davies review (2019), recent evidence supports the trends of varicose vein case rates of 51.9 cases per 1000 women and 39.4 cases per 1000 men. It also shows that the prevalence of CVD and varicose veins vary widely by region, though they are highest in Western countries [1]. A comprehensive review evaluated articles published in the English language over more than 55 years, and revealed prevalence estimates for varicose veins of < 1 to 73% in women and 2 to 56% in men. The reported wide ranges in prevalence estimations reflect differences in the population distribution of risk factors, accuracy in application of diagnostic criteria, and the quality and availability of medical diagnostic and treatment resources [4]. In Egypt, Aly et al. cross-sectional study (2020), conducted on women, found that 51.1% of women (aged 15-55 years old) had varicose veins [5].

Previous studies described the non-modifiable factors associated with LLVV such as positive family history of varices, increasing age, woman gender, and pregnancy [4, 6]. On the other hand, the association between modifiable factors such as life style and occupational factors and the development of LLVV were debated. There is an ongoing controversy as to whether obesity is a primary risk factor for LLVV, or it acts as an aggravating factor only [7]. In addition, few studies examined the effect of estrogen therapy, hormone replacement therapy, physical activity, hypertension, diabetes mellitus, and traumatic injury to the extremities on LLVV [8, 9].

Low dietary fiber intake, irregular defecation, and straining at initial bowel movements have been suggested as risk factors for LLVV, but the findings have been inconsistent [4, 6]. In addition, effect of smoking habit and smoking intensity on the development of LLVV is debated [10–12]. Smoking may affect veins through oxidative stress, hypoxia (through carbon monoxide and nitric oxide fixation to hemoglobin), and endothelial damage; however, mechanisms leading to tissue damage are uncertain [13].

Occupational mechanical exposure in term of prolonged standing, sitting, walking, and lifting heavy objects have also been examined in a number of studies, of which some found a positive association with LLVV [4, 6], while other did not [8]. A study goes even so far to suggest a reverse relationship between prolonged sitting posture and prevalence of LLVVs, while confirming the impact of a prolonged standing posture of the occurrence of LLVV [4].

In Egypt, although venous diseases are one of the most common medical problems, there is few documented data about risk factors for LLVV among Egyptian population; those studies were done long time ago [14, 15]. It was essential to update data; therefore, the current study was conducted to study the epidemiological, life style, and occupational factors associated with LLVV among cases attending vascular surgery clinic at the Alexandria Main University Hospital, Egypt, as determining the modifiable risk factors would be helpful in establishing an evidence-based strategy for prevention of LLVV among Egyptian population.

## Methods

### Research design and setting

A case control study was conducted at the vascular surgery outpatient clinic and ophthalmology outpatient clinic at the Alexandria Main University Hospital from January through May 2019. The field work was conducted over a 5-month period, two visits per week at each outpatient clinic.

### Participants

#### Selection of cases

Cases attending the vascular surgery outpatient clinic during the period of the study were examined by a consultant of vascular surgery to identify cases meeting the case definition of LLVV. The case definition includes dilated, tortuous, and palpable subcutaneous veins typically larger than 3-4 mm in diameter mostly in the legs and ankles [16]. Cases of LLVV were diagnosed and classified using the Clinical, Etiologic, Anatomic, Pathophysiologic classification system of chronic venous insufficiently (CEAP) [17], which is widely accepted in the clinical and scientific communities. The classification was developed in an effort to incorporate use of duplex scanning in the diagnosis, and standardize evaluation for comparison of outcomes across clinical studies. CEAP includes clinical symptoms such as pain, presence of varicose veins, edema, hyperpigmentation, and ulcer. It also considers the etiology (primary or secondary), anatomic distribution and position, pathogenic mechanism (venous reflux, obstruction, or both), and produces a score based on the severity of the disease [17]. During the study period, a total of 168 cases meeting the diagnostic criteria were examined, of whom 150 agreed to participate in the study.

#### Selection of controls

An equal number of controls (*n* = 150) was selected from the ophthalmology outpatient clinic of the same hospital. The same procedure (CEAP) was used to exclude the presence of LLVV by the same consultant of vascular surgery. In the current study, patients with past history of thrombosis, or refused to participate in the study were excluded.

#### Power analysis

A power analysis was conducted using the Open Source Epidemiologic Statistics for Public Health (OpenEpi) [18] to ensure that the number of identified cases will detect a difference between cases and controls. The power analysis showed that enrolling 150 cases of LLVVs and 150 controls is capable to detect the least difference in the probability of exposure between cases and controls of 14% in respect to major risk factors, with an expected odds ratio of 2.2 at a power of 81.36% and confidence level of 0.95 (α = 0.05).

### Research tools

Data was collected from cases and controls using a pre-designed and pre-tested interview questionnaire and clinical examination.

#### Interview questionnaire was conducted to collect the following data

##### Sociodemographic data

Including participants’ age, sex, rural/urban residence, education attainment, and marital status.

##### Epidemiological and life style factors

- ***Level of activity***. *Regular physical exercise* (exercising for 150 min of moderate intensity aerobic physical activity throughout the week as running, walking, swimming, or spinning) [19]; *frequency of lifting heavy objects/day*; *standing hours/day*; *sleeping hours/day*; *and sitting hours/day*.
- ***Smoking habit***. Inquiry was made into smoking habit and participants were classified into current smoker (still smoking at least one cigarette daily for as long as 1 year), ex-smoker (quit smoking for at least 6 months prior to the date of the study), and non-smoker (never smoked or smoked less than one pack per month or 20 packs in his/her whole life) [11]. Among smokers, *smoking index* (*SI*) was calculated by multiplying the number of cigarettes smoked per day by long life duration of smoking in years. Smokers were classified according to their SI into light smokers (SI < 200), moderate smokers (SI = 200-400), and heavy smokers (SI > 400) [20].
- ***Dietary habits and intestinal motility***. *Frequency of eating diet rich in fibers* (fruit, dark green vegetables, orange vegetables, legumes, and whole grains); *drinking water/day*; *regularity of defecation times/day*; *posture while defecation* (*sitting/squatting*).
- ***Reproductive history of married women***. *Gravidity*; *parity*; and *the use of oral contraceptives* (*OCs*).
- ***Family and medical history***. *Positive family history of varices* (*LLVV*, *hemorrhoids*, *varicocele*) among first degree relatives (parents, full siblings, or children) and second degree relatives (grandparents, grand-children, aunts, uncles, nephews, nieces, or half siblings); *history of immobilization for 1-3 months*; and *history of leg trauma* (sprains, strains, fractures, or joint dislocation).

##### Occupational factors

- ***Employment status***. Participants were categorized into two categories: ever and never employed. The ever employed includes participants who are currently employed, retired, or those with past occupations. Ever employed participants were classified according to the International Standard Classification of Occupations (ISCO) [21].
- ***Duration of employment in years***.
- ***Working hours/day and working hours/week****.*
- ***Work time*** (daytime or shift work)
- ***Nature of work***. Participants were classified into white collar (performing professional, managerial, or administrative work), pink collar (related to customer interaction, entertainment, sales, or other service oriented work), and blue collar (job requires manual labor) [22].
- ***Posture at work****.* Spending more than half their lifetime working hours engaged in particular posture (standing, sitting, walking, and lifting heavy objects).

### Clinical examination was conducted to collect the following data:

#### Anthropometric measurements

Weight and height was obtained following standard procedures, and *body mass index* (*BMI*) was computed to classify participants into underweight (BMI < 18 kg/m^2^), normal weight (BMI from 18.5 to < 25kg/m^2^), overweight (BMI from 25 to < 30kg/m^2^), and obese (BMI ≥ 30 kg/m^2^) [23].

#### Clinical examination of lower limbs

Participants were subjected to a clinical examination of the lower limbs by a consultant of vascular surgery to ascertain or exclude the presence of LLVVs. Cases were classified into six categories following the CEAP system [17] including: C_0_, no visible or palpable signs of cardiovascular disease (CVD); C_1_, telangiectasia or reticular veins; C_2_, varicose vein > 4 mm in diameter; C_3_, edema as a sequel of varicose vein; C_4_, skin changes (pigmentations, venous eczema, etc.); C_5_, skin changes with healed ulcerations; and C_6_, skin changes with active ulcerations [17].

### Statistical analysis 

The SPSS v.20 (IBM Corp. Released 2011. IBM SPSS Statistics for Mac, Armonk, NY, USA) was used for data entry and analysis. Data was described using number and percentages as well as mean and standard deviation. Univariate logistic regression analysis was done to compute the odds ratio (OR) and the associated 95% confidence interval (95% CI) to quantify the risk of LLVVs associated with the potential related factors.

Multivariate logistic regression analysis was conducted to model LLVVs as a function of sociodemographic, epidemiologic, life style, and occupational risk factors. As occupational risk factors were applicable only to ever employed participants (*n* = 234), two multivariate regression models were constructed. The first one was a stepwise multivariate logistic regression analysis including all participants (*n* = 300), in which the potential epidemiological and life style risk factors that were significant in univariate analysis were considered in addition to age, gender, employment status, and family history of varices. The second model was a stepwise model limited to the ever employed participants (*n* = 234) in which the aforementioned factors were considered in addition to the potential occupational risk factors to study their independent effect.

Significance of the obtained results was judged at the 5% level (α = 0.05). The explained variance of logistic regression models was determined by the Nagelkerke’s R^2^ and Hosmer and Lemeshow goodness-of-fit test. Significant factors in univariate analysis that include cells having a count less than 5 were excluded from the multivariate analyses to avoid distortion of the model.

## Results

According to CEAP classification system, the majority of cases with LLVV were categorized as C2 (72.0%, *n* = 108) and C3 (28.0%, *n* = 42). In addition, cases with LLVV reported symptoms such as pain (96.0%, *n* = 144), lower limb edema (27.3%, *n* = 41), while bleeding or ulcers were not reported. Among cases, 82.7% have received conservative treatment, 24.7% reported frequent leg raising to alleviate symptoms, and 14% reported frequent wearing compression stoking.

Univariate analysis revealed insignificant differences in the age and sex distribution of cases and controls. The mean age of cases was 49.90 ± 12.09 years compared with 50.26 ± 10.98 years among controls (*p* = 0.78). However, cases were 4 times more likely to report never been to school or completed their basic education compared with controls (OR = 4.07; 95% CI = 2.51, 6.62) (Table 1).

**Table 1:** Sociodemographic characteristics of studied patients attending the vascular surgery and ophthalmology outpatient clinics at the Alexandria Main University Hospital, 2019, Egypt

Sociodemographic characteristics	Cases (***n*** = 150)		Controls (***n*** = 150)		OR (95% CI)	*P* value
	No.	%	No.	%		
Gender						
Male^^^	68	45.3	74	49.3	1.17 (0.74, 1.84)	0.48
Female	82	54.7	76	50.7		
Residence						
Rural^^^	13	8.7	5	3.3	0.36 (0.12, 1.04)	0.05*
Urban	137	91.3	145	96.7		
Level of education						
Unschooling/basic education	108	72.0	58	38.7	4.07 (2.51, 6.62)	>0.001***
Secondary/higher education^^^	42	28.0	92	61.3		
Marital status						
Never married^^^	4	2.7	5	3.3	1	
Married	111	74.0	121	80.7	1.14 (0.30, 4.37)	0.84
Widowed	19	12.7	16	10.7	1.48 (0.34, 6.47)	0.59
Divorced	16	10.7	8	5.3	2.50 (052, 11.9)	0.25
Age (years)						
Min-max	25-70		28-75			
Mean ± SD	49.90 ± 12.09		50.26 ± 10.98		0.99 (0.98, 1.02)	0.78

*SD* standard deviation, *OR* odds ratio, *CI* confidence interval

^^^Reference category

*p≤0.05

***p>0.001

Cases of LLVVs were 45.34 times more likely to report lifting heavy objects (OR = 45.34; 95% CI = 6.12, 345.94). Cases were significantly more likely to report standing for more than 4 h daily (OR = 6.95; 95% CI = 4.08, 11.86) and to sleep less than 8 h per day (OR = 5.51; 95% CI = 3.02, 10.04). No excess risk was found between cases and neither controls in respect to exercising nor the number of hours spent sitting each day (Table 2).

**Table 2:** Epidemiological and life style factors associated with lower limbs varicose veins (LLVV)

Epidemiological and life style factors	Cases (***n*** = 150)		Controls (***n*** = 150)		OR (95% CI)	*P* value
	No.	%	No.	%		
*Level of activity*						
Exercising						
Yes (regular/irregular)^&^^	27	18.0	29	19.3	1.09 (0.61, 1.95)	0.76
No	123	82.0	121	80.7		
Lifting heavy objects (frequency/day)						
Frequent/very frequent^$^	35	23.3	1	0.7	45.34 (6.12, 345.94)	>0.001***
Occasional/rare/never^	115	76.7	149	99.3		
Standing hours/day						
> 4 h/day	89	59.3	26	17.3	6.95 (4.08, 11.86)	>0.001***
≤ 4 h/day^	61	40.7	124	82.7		
Sleeping hours/day						
< 8 h/day	62	41.3	17	11.3	5.51 (3.02, 10.04)	>0.001***
≥ 8 h/day^	88	58.7	133	88.7		
Sitting hours/day						
< 8 hours/day	77	51.3	72	48.0	1.14 (0.72, 1.79)	0.64
≥ 8 hours/day^^^	73	48.7	78	52.0		
*Anthropometric measurements*						
Body mass index (BMI)						
Underweight and normal (BMI < 25)^^^	8	5.3	19	12.7	2.57 (1.09, 6.08)	0.02*
Overweight and obese (BMI ≥ 25)	142	94.7	131	87.3		
Min-max	21.60-48.47		21.48-39.45			
Mean ± SD	31.19 ± 4.71		27.68 ± 2.57		1.32 (1.22, 1.44)	>0.001***
*Smoking*						
Smoking status						
Smoker	45	30.0	27	18.0	1.95 (1.13, 3.36)	0.01*
Ex-smoker/non-smoker^^^	105	70.0	123	82.0		
Smoking intensity^#^						
Light smoker^^^	34	63.0	32	91.4	6.27 (1.70, 23.16)	>0.01**
Moderate/heavy smoker	20	37.0	3	8.6		
Smoking index (SI)						
Min-max	20-500		25-300			
Mean ± SD	188.5 ± 103.6		141.8 ± 73.4		1.006 (1.001, 1.01)	0.02*
*Dietary habits and intestinal motility*						
Consumption of diet rich in fibers						
Occasional/rare/never	103	68.7	30	20.0	8.76 (5.16, 14.86)	>0.001***
Frequent/very frequent^^^	47	31.3	120	80.0		
Drinking water (cups/day)						
≥ 5 cups/day	136	90.7	60	40.0	14.57 (7.68, 27.62)	>0.001***
≥ 5 cups/day^^^	14	9.3	90	60.0		
Regularity of defecation						
Irregular	97	64.7	40	26.7	5.03 (3.07, 8.24)	>0.001***
Regular^^^	53	35.3	110	73.3		
Posture while defecation						
Squatting	25	16.7	2	1.3	14.80 (3.43, 63.71)	>0.001***
Sitting^^^	125	83.3	14	98.7		
Taking laxatives						
Yes	31	20.7	7	4.7	5.32 (2.26, 12.51)	>0.001***
No^^^	119	79.3	143	95.3		
*Medical and family history*						
Family history of varices						
Positive family history	58	38.7	40	26.7	1.73 (1.06, 2.82)	0.02*
Negative family history/not known^^^	92	61.3	110	73.3		
Immobilization for 1-3 months						
Yes	13	8.7	0	0.0		
No^^^	137	91.3	150	100.0		
Previous leg trauma						
Yes	9	6.0	1	0.7	9.51 (1.19, 76.03)	0.01*
No^^^	141	94.0	149	99.3		
*Reproductive factors*	(*n* = 81)	(*n* = 75)				
Gravidity^a^						
≥ 4	36	44.4	12	16.0	4.20 (1.97, 8.95)	>0.001***
< 4^^^	45	55.6	63	84.0		
Parity^a^						
≥ 4	32	39.5	6	8.0	7.51 (2.91, 19.33)	>0.001***
< 4^^^	49	60.5	69	92.0		
Use of OCs^a^						
Yes (regular/irregular)	36	44.4	10	13.2	5.20 (2.34-11.53)	>0.001***
No^^^	45	55.6	65	86.8		
Duration of OCs use (years)^b^						
< 5 years^^^	5	13.9	7	70.0	14.46 (2.77, 75.3)	0.001**
≥ 5 years	31	86.1	3	30.0		
Min-max	1-12		1-10			
Mean ± SD	6.91 ± 3.21		3.30 ± 2.83		1.47 (1.10, 1.97)	>0.01**

Abbreviations: *SI* smoking index, *SD* standard deviation, *OR* odds ratio, *CI* confidence interval, *OCs* oral contraceptive pills

^^^Reference category

^#^Among smokers (cases *n* = 54, controls *n* = 35), light smokers (SI < 200), moderate/heavy smokers (SI ≥ 200)

^$^Where frequent is ≥ 5 times per week

^&^Regular exercise defined as 150 min of moderate intensity aerobic physical activity per week

^a^Number of women excluding single women (cases *n* = 81, controls *n* = 75)

^b^Number of women using OCs (cases *n* = 36, controls *n* = 10)

*p≤0.05

**p>0.01

***p>0.001

An excess of smokers was encountered among cases of LLVV compared with controls (30.0% compared with 18.0%) resulting in an estimated risk of 1.95 (95% CI = 1.13, 3.36). Smokers with LLVV were 6.27 times more likely to be moderate or heavy smokers (95% CI = 1.70, 23.16) and have a significantly higher smoking index (OR = 1.006; 95% CI = 1.001, 1.01) (Table 2).

Relative to controls, cases with LLVVs were significantly more likely to report never or infrequently consuming diet rich in fibers (OR = 8.76; 95% CI = 5.16, 14.86), drink less than 5 cups of water/day (OR = 14.57; 95% CI = 7.68, 27.62), and have irregular defecation habit (OR = 5.03; 95% CI = 3.07, 8.24). Compared with controls, a significantly higher percentage of cases with LLVVs reported squatting posture during defecation (16.7% compared to 1.3%) and taking laxatives (20.7% compared to 4.7%). Cases were 14 times more likely to squat during defecation (OR = 14.80; 95% CI = 3.43, 63.71) and 5 times more likely to take laxatives (OR = 5.32; 95% CI = 2.26, 12.51). Cases with LLVV were significantly more likely to be overweight or obese (BMI ≥ 25 kg/m^2^) than controls (OR = 2.57; 95% CI = 1.09-6.08) (Table 2).

Reproductive factors were examined among married, widowed, and divorced women in the current study (cases *n* = 81, controls *n* = 75); 70.4% of women with LLVV reported having the disease after the first pregnancy. Women with LLVV were significantly more likely to have high gravidity (≥ 4 times) (OR = 4.2; 95% CI = 1.97, 8.95), and to have high parity (≥ 4 times) (OR = 7.51; 95% CI = 2.91, 19.33) than controls. Furthermore, women with LLVV tended to use OCs 5.20 times higher than controls (95% CI, 2.34-11.53). Among OCs users (cases *n* = 36, controls *n* = 10), the mean duration of use of OCs was significantly higher among cases with LLVV (6.91 ± 3.21) compared with controls (3.30 ± 2.83) (OR = 1.47; 95% CI = 1.10, 1.97) (Table 2).

Regarding family and medical history, cases with LLVV tended to have a positive family history of varices 1.73 times higher than controls (95% CI = 1.06, 2.82); they reported positive family history of LLVV, varicocele, and hemorrhoids (87.9%, 6.9%, and 5.2% respectively). Furthermore, cases with LLVV were significantly more likely to have a history of leg trauma than controls (OR = 9.51; 95% CI = 1.19, 76.03) (Table 2).

Cases and controls were comparable in respect to their employment status (*p* = 0.78). Among ever employed participants (cases *n* = 118 and controls *n* = 116); the majority of cases with LLVV were plant or machine operators (38.1%) and craft workers (27.1%), while the majority of controls were technicians/associated professionals (37.1%) and service/sales workers (18.1%) (Table 3).

**Table 3:** Occupational factors associated with lower limbs varicose veins (LLVV)

Occupational factors	Cases		Controls		OR (95% CI)	*P* value
	No.	%	No.	%		
*Employment status*	(*n* = 150)		(*n* = 150)			
Never employed	32	21.3	34	22.7	1.08 (0.62, 1.86)	0.78
Ever employed (currently/previously)	118	78.7	116	77.3		
*Among ever employed*	(*n* = 118)		(*n* = 116)			
Duration of employment (years)						
Min-max	2-50		2-50			
Mean± SD	23.55 ± 13.70		24.50 ± 14.09		0.99 (0.98, 1.01)	0.60
ISCO classification^#^						
Professionals^^^	3	2.5	22	19.0	1	
Technicians and associated professionals	24	20.3	43	37.1	0.27 (0.01, 4.00)	0.34
Service and sales workers	10	8.5	21	18.1	1.11 (0.09, 12.96)	0.93
Skilled agricultural and fishery workers	3	2.5	2	1.7	0.95 (0.07, 11.78)	0.97
Craft and related trade workers	32	27.1	8	6.9	3.00 (0.15, 59.89)	0.47
Plant and machine operators	45	38.1	18	15.5	8.00 (0.64, 99.66)	0.10
Elementary occupations	1	0.8	2	1.7	5.00 (0.42, 58.63)	0.20
Working hours/day^#^						
Working > 8 h/day	47	39.8	22	19.0	2.82 (1.56, 5.11)	>0.001***
Working ≤ 8 h/day^^^	71	60.2	94	81.0		
Working hours/week^#^						
Working > 48 h/week	47	39.8	24	20.7	2.53 (1.41, 4.53)	>0.01**
Working ≤ 48 h/week^^^	71	60.2	92	79.3		
Work time^#^						
Day work^^^	75	63.6	115	99.1	65.93 (8.8, 489.1)	0.001**
Shift work	43	36.4	1	0.9		
Nature of work^#^						
Blue/pink collar	91	77.1	53	45.7	4.00 (2.28, 7.04)	>0.001***
White collar^^^	27	22.9	63	54.3		
Posture at work^#^						
Standing	66	55.9	18	15.5	6.91 (3.71, 12.84)	>0.001***
Sitting^^^	52	44.1	98	84.5		
Sitting hours/working day^#^						
Sitting ≤ 4 h/ working day	65	55.1	21	18.1	5.54 (3.05, 10.06)	>0.001***
Sitting < 4 h/ working day^^^	53	44.9	95	81.9		
Standing hours/working day^#^						
Standing <4 h/working day	64	54.2	18	15.5	6.45 (3.47, 11.98)	>0.001***
Standing >4 h/working day^^^	54	45.8	98	84.5		
Lifting heavy objects (hours/working day)^#^						
≥ 2 h/working day	7	5.9	1	0.9	7.25 (0.87, 59.90)	0.06
< 2 h/working day^^^	111	94.1	115	99.1		

*ISCO* International Standard Classification of Occupations, *SD* standard deviation, *CI* confidence interval, *OR* odds ratio

^^^Reference category

^#^Number of participants currently/previously employed (cases = 118, controls = 116)

**p> 0.01

***p>0.001

Cases with LLVV were significantly more likely to work more than 8 h/day than controls (OR = 2.82; 95% CI = 1.56, 5.11), and to work more than 48 h/week than controls (OR = 2.53; 95% CI = 1.41, 4.53). In addition, cases with LLVV tended to have shift work 65.93 times higher than controls (OR = 65.93; 95% CI = 8.88, 489.06), and to be a blue/pink collar 4 times higher than controls (OR = 4.00; 95% CI = 2.28, 7.04). Regarding posture at work, cases with LLVV were significantly more likely to stand at work for more than 4 h/working day than controls (OR = 6.45; 95% CI = 3.47, 11.98), and to sit at work for equal to or less than 4 h/working day than controls (OR = 5.54; 95% CI = 3.05, 10.06) (Table 3).

According to the stepwise logistic regression analysis conducted among all participants (*n* = 300) in the current study, the independent predictors of LLVVs were as follows: frequent lifting of heavy objects (OR = 59.79; 95% CI = 6.01, 584.36), drinking < 5 cups of water/day (OR = 6.95; 95% CI = 2.78, 17.33), infrequent/no consumption of fiber-rich food (OR = 4.27; 95% CI = 1.95, 9.37), standing more than 4 h per day (OR = 3.65; 95% CI = 1.63, 8.17), irregular defecation habit (OR = 3.34; 95% CI = 1.68, 6.60), sleeping less than 8 h per day (OR = 2.86; 95% CI = 1.14, 7.16), smoking (OR = 2.53; 95% CI = 1.15, 5.58), and age (OR =1.05; 95% CI = 1.02, 1.09). Those factors were adjusted for gender, employment status, and family history of varices. This model was able to correctly classify 87.0% of cases and 81.0% of controls in the current study (Table 4).

**Table 4:** Stepwise logistic regression analysis of the independent predictors of lower limb varicose veins (LLVV) among all participants (*n* = 300)

Independent predictors	Coefficient	Adjusted OR^**†**^	95% CI	*P* value
Age	0.05	1.05	(1.02, 1.09)	< 0.01**
Smoking	0.93	2.53	(1.15, 5.58)	0.02*
Infrequent/no consumption of fiber-rich food	1.45	4.27	(1.95, 9.37)	< 0.001***
Drinking < 5 cups of water/day	1.94	6.95	(2.78, 17.33)	< 0.001***
Irregular defecation habit	1.20	3.34	(1.68, 6.60)	< 0.01**
Frequent lifting of heavy objects/day	4.09	59.79	(6.01, 584.36)	< 0.001***
Standing > 4 h/day	1.29	3.65	(1.63, 8.17)	< 0.01**
Sleeping < 8 h/day	1.05	2.86	(1.14, 7.16)	0.02*

Abbreviations: *OR* odds ratio, *CI* confidence interval

Model X^2^ = 200.91 (p < 0.001); Nagelkerke’s R^2^ = 0.65; Cox and Snell R^2^ = 0.48; Hosmer and Lemeshow X^2^ = 5.57 (p=0.69)

^†^OR adjusted for gender, employment status, family history of varices, and potential life style risk factors significant in univariate analysis (posture during defecation, taking laxatives, and body mass index)

Occupational risk factors and significant factors that include cells having a count less than 5 were excluded from the regression model

*p<0.05

**p<0.01

***p<0.001

Among ever employed participants (*n* = 234), the stepwise logistic regression model, which aimed at studying the independent effect of occupational risk factors, revealed that standing posture at work was an independent predictor of LLVV (OR = 3.10; 95% CI = 1.02; 9.38), in addition to other epidemiological and life style predictors. This model was able to correctly classify 87.3% of the cases and 82.0% of the controls in the current study (Table 5).

**Table 5:** Stepwise logistic regression analysis of the independent predictors of lower limb varicose veins (LLVV) among ever employed participants (*n* = 234)

Independent predictors	Coefficient	Adjusted OR^**†**^	95% CI	*P* value
Standing posture at work	1.13	3.10	(1.02, 9.38)	0.04*
Age	0.06	1.06	(1.02, 1.10)	<0.01**
Frequent lifting of heavy objects/day	3.63	37.59	(3.52, 400.86)	<0.01**
Standing > 4 h/day	1.26	3.52	(1.20, 10.33)	0.02*
Infrequent/no consumption of fiber-rich food	1.45	4.24	(1.70, 10.62)	<0.01**
Drinking < 5 cups of water/day	2.24	9.42	(3.43, 25.89)	<0.001***
Irregular defecation habit	1.87	6.46	(2.73, 15.27)	<0.001***
Taking laxatives	−1.63	0.20	(0.05, 0.08)	0.02*

Abbreviations: *OR* odds ratio, *CI* confidence interval

Model X^2^ = 157.19 (p < 0.001); Nagelkerke’s R^2^ = 0.65; Cox and Snell R^2^ = 0.48; Hosmer and Lemeshow X^2^ = 10.48 (*p* = 0.23)

^†^OR adjusted for gender, family history of varices, and potential occupational and life style risk factors significant in univariate analysis (working hours per week, nature of work, standing hours/working day, sitting hours/working day, body mass index, smoking, posture during defecation, and sleeping hours/day)

Significant factors that include cells having a count less than 5 were excluded from the regression model

*p<0.05

**p<0.01

***p<0.001

## Discussion

The current study identified important modifiable life style and occupational risk factors for the development of LLVV. Frequent lifting of heavy objects, drinking < 5 cups of water/day, infrequent/no consumption of fiber-rich food, standing more than 4 h/day, irregular defecation habit, sleeping less than 8 h/day, and smoking were independent predictors of LLVV. In addition, standing posture at work was found to be an independent occupational risk factor for LLVV among ever employed participants.

Age was found to be an independent predictor of LLVV in the current study; an effect that was masked by other factors in univariate analysis, whereas gender and family history of varices were not associated with risk of LLVV. Similarly, several studies revealed an association between increasing age and varicose veins [10–12, 24, 25], no sex-dependent difference in LLVV, and a negative relation between family history and varicose veins [12]. On the contrary, in Ziegler et al. study, age was not a risk factor for varicose veins [26]. Also, some studies reported association between positive family history and varicose veins [6, 27]; the results of studies must be interpreted with caution, as varicose veins are a common problem, thus, a large proportion of study subjects would report a positive family history.

Besides, some studies revealed an association between female gender and varicose veins [10, 11, 24, 26]. Different distribution of reproductive risk factors among women in different studies might lead to diverse results. Gravidity, parity, and use of OCs could independently increase the risk of LLVV among women compared with men, in some studies. Different findings may also be due to different study designs, sample size, and population characteristics.

The current study as well as previous studies reported the higher risk of LLVVs associated with high parity [4, 6, 12, 25] and the use of OCs [28]. Beebe-Dimmer et al. [4] attributed the adverse effects of pregnancy to the increase in blood volume as well as the increase in intra-abdominal pressure and central venous return resulting from fetal growth and weight gain; the elevated pressure can result in valve failure and progression of varices. Also, the increase in the levels of relaxin, estrogen, and progesterone during pregnancy play a role in the development of varices. In contrast, other studies did not support the association of LLVVS with pregnancy [28] or the use of hormonal contraceptives [12, 26]. The variation in the effect of OCs between this study and that of Yun et al. study [12] could be attributed to the difference in the distribution of reproductive risk factors; all of the women in their study were working nurses and most of them were unmarried. In the current research, the small number of ever married women and those who ever used hormonal contraceptives precluded studying the independent effect of reproductive factors.

In the current study, never going to school or obtaining only basic education, which is a proxy for low socioeconomic status, was significantly associated with LLVV. This could be explained by the fact that level of education determines the nature of occupation (blue/pink collar vs white collar) which directly affects the development of varices. Among ever employed participants in the current study, 65% of cases with LLVV were plant and machine operators and craft workers, while professionals represented 2.5% of cases. Likewise, in other studies, the majority of cases were manual laborers, farmers, house wives, nurses, utility workers, and cleaners [24, 26, 29]. This supports the observation that occupation involving violent muscular activity are at high risk for developing varicose veins. Hot and humid workplace conditions and socioeconomic may also be implicated [26].

Among ever employed participants in the current study, standing posture at work was found to be an independent predictor of LLVV. Most studies indicate that working in a position resulting in protracted orthostasis may increase the prevalence and severity of LLVV; a significantly higher prevalence of varicose veins was found in employees working in a prolonged standing posture [6, 24, 26, 30]; while the lowest prevalence was found in medical technician assistants, secretaries, and scientific staff, since these professions have the shortest mean periods of standing at work [26].

Regarding physical activity, frequent lifting of heavy objects, standing for more than 4 h/day, and sleeping for less than 8 h/day, were found to be independent predictors of LLVV in the present study and in Tabatabaeifar et al. study [25]. Whereas no relation was found between regular physical exercise and the development of LLVV in the current study and in Yun et al. study [12]. On the contrary, in other studies, regular exercising was found to be a protective factor [10, 24]. The difference in findings might be due to different definitions of the regular exercising term in studies.

As regard dietary habits and intestinal motility, low dietary fiber intake, drinking less than 5 cups of water/day, and irregular defecation were independent predictors of LLVV in the present study and in other studies [4, 24, 31] which proposed that constipation and increased intra-abdominal pressure contributed to obstruction of venous return. People usually take laxatives to regulate their irregular bowel movement; this explains the change in findings in the present study, where taking laxatives was a risk factor for LLVV in univariate analysis, while in multivariate analysis, it had a protective effect.

Overweight or obesity (BMI ≥ 25) was not a predictor of LLVV in the current study. In Seidel et al. study, the association between obesity and varicose veins was found in women but not in men [8]; however, in his study, there was an observed gender difference, besides, parity may act as a confounding factor due to the fact that parous women tend to have high average body weight [8]. In literature, the data on correlation between obesity and varicose veins is controversial; and whether it has an independent or an aggravating effect on the development of varicose veins is still debatable [7].

Smoking was an independent predictor of LLVV in the current study; smokers had 2.53 times greater risk to develop LLVV. Moreover, smoking intensity was significantly associated with LLVV; moderate/heavy smokers had 6.27 times greater risk to develop LLVV. Similarly, in a multicenter review and in other studies, the proportion of varicose veins cases with history of smoking was 19.4%, and 45.6% [29], smokers had 1.8 times greater risk [30], and smoking pack-years significantly increases the odds of varicose veins by 1.12 [10]. It has been hypothesized that smoking leads to hypoxia, production of proinflammatory factors within the vessel wall, biochemical modifications on the venous endothelium that increases the vasomotor tonicity in the venous walls, and lengthening of scarring time which influence the trophic disorders associated with LLVV [30].

Few participants in the present study had a history of leg trauma and long immobilization which was significantly associated with LLVV in accordance with Abelyan et al. study [10]. In contrast, Yun et al. study found no significant association between leg injury and varicose veins [12]. The independent effect of those variables calls for further studies on a larger sample.

### Limitations of the study

The current case control study (retrospective study) might be subjected to recall bias; cases with LLVVs would probably be more aware of the past medical and family events related to their conditions. Additionally, the relatively small number of ever married women limited studying the independent effect of reproductive factors.

## Conclusion

The present study highlighted seven *modifiable* independent predictors of LLVV mostly related to the life style. These findings provide a foundation for planning an evidence-based low-cost strategy for prevention of LLVV among Egyptian population. Future studies are recommended to examine cause-effect relationship, and evaluate independent effect of reproductive factors on the development of LLVV.

## Data Availability

Data and materials are available on reasonable request. Confidentiality and security of data and materials were insured through all stages of the study.

## Abbreviations

BMI: Body mass index
CEAP: Clinical, Etiologic, Anatomic, Pathophysiologic
CI: Confidence interval
CVD: Chronic venous disease
ISCO: International Standard Classification of Occupations
LLVV: Lower limb varicose veins
OCs: Oral contraceptive pills
OR: Odds ratio
SD: Standard deviation
SI: Smoking index
